# Investigation of a source model for a new electronic brachytherapy tandem by film measurement

**DOI:** 10.1002/acm2.12440

**Published:** 2018-08-13

**Authors:** Elijah Martin, Keith Sowards, Brian Wang

**Affiliations:** ^1^ Department of Radiation Oncology University of Louisville Louisville KY USA

**Keywords:** electronic brachytherapy, radiochromic film

## Abstract

**Purpose:**

To investigate the accuracy of a vendor‐supplied source model for a new Xoft Axxent 0‐degree titanium tandem by film measurement.

**Methods:**

We measured the anisotropy factors at varying distances and angles from the tandem in water using radiochromic film (Gafchromic EBT3) and an Epson Perfection v750 desktop flatbed scanner (US Epson, Long Beach, CA). A 0‐degree tandem was placed vertically in a water phantom. Four pieces of film, each at varying depths, were positioned orthogonal to the longitudinal axis of the tandem for azimuthal anisotropy measurements. Polar anisotropy measurements were taken with the film aligned parallel to the tandem. An absolute dose calibration for the film was verified with a PTW 34013 Soft X‐Ray Chamber. The film measurements were analyzed using different color channels. The measured polar anisotropy for varying source positions was compared to the vendor's data. Azimuthal anisotropy was measured as a function of the radius and angle, and normalized to the mean value over all angles at the specified radius.

**Results:**

The azimuthal anisotropy of the tandem and source was found to be consistent for different positions along the tandem's longitudinal axis and at varying distances from the tandem. Absolute dose using a calibrated parallel plate chamber showed agreement to within 2% of expected TPS values. The custom tandem, which has a thicker tip than the wall, was attenuating the 50 kV photons more than expected, at the angles where the photons had more wall material to traverse. This discrepancy was verified at different distances from the tandem and with different measurement techniques. As distance increased, anisotropy values had better agreement.

**Conclusions:**

We quantified the agreement between the measured and provided anisotropy factors for a new Xoft Axxent 0‐degree titanium tandem. Radiochromic film response at low kV energy was also investigated. Our results showed that vendor‐supplied TG‐43 values were appropriate for clinical use at majority of the angles. A rigorous quality assurance method for new electronic brachytherapy sources and applicators, along with complete knowledge of all dosimetric measuring tools, should be implemented for all parts of the verification and commissioning process.

## 1 INTRODUCTION

Gynecological cancers are one of the most prevalent cancers in women, with an estimated 107,470 new cases diagnosed and 31,000 deaths in 2017.^1^ A large portion of these cancers are treated with high‐dose rate (HDR) procedures, such as an Iridium‐192 source placed within a tandem and a set of ovoids, to deliver dose to high‐risk volumes. The electronic brachytherapy device, manufactured by Xoft, Inc. (a subsidiary of iCad, Inc., Nashua, NH) utilizes a miniaturized x‐ray tube to generate x rays up to 50 keV. This proprietary system has been used to treat breast, skin, and gynecological cancers.^2^, ^3^, ^4^ Delivering an isotope‐free treatment has many benefits, including minimal shielding and no need for a radioactive license.^5^ Recently, Xoft Inc. has received FDA clearance to treat cervical cancers using their tandem and ovoid systems.

### 1.A Source model

The miniature electronic 50 kVp x‐ray source, model S700, and its properties have been studied and quantized by Rivard et al.^6^ Modifications to the S700 source design were implemented by the manufacturer and characterized by Hiatt et al.^7^ Photon energy spectra were published by Hiatt et al. for making detector response corrections for dosimetric measurements.^7^ An extended source, 50 cm in length and capable of reaching the tip of the tandem, is manufactured exclusively for the cervical applicators and differ from the 25 cm source only in length. Liu et al. concluded that the change in beam quality due to source variation and source aging is significant and each source should be treated on an individual basis.^8^ Independent verification and validation of all equipment and software provided by vendor is of upmost importance for accuracy of treatment and patient safety. The primary objective of our study is to characterize this new source type by measurements.

### 1.B Applicators design

**Figure 1:**
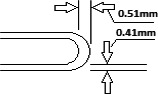
Cross‐section of 0 degree tandem.

The cervical applicators provided by Xoft Inc. are based on the Henschke‐style applicator. There are four tandem geometries; 0°, 15°, 30°, and 45°, and two colpostat (ovoids) geometries; 0°, 15°. The colpostat's also are available in varying diameters of 2.0, 2.5, and 3.0 cm. The walls of the applicators are constructed of 0.41 mm titanium, and the dome is constructed of 0.51 mm of titanium (Fig. 1). The thickness difference between the wall and dome is to attenuate more photons and minimize the forward dose. As the source is successively pulled back in the tube, the wall thickness for the oblique (nonorthogonal) x rays will be subject to additional attenuation and beam hardening from the tube wall. This increased material will both attenuate the dose and harden the x rays off‐axis. A dwell position‐dependent anisotropy function is required for the first few dwell positions in the tandem.^9^ Xoft Inc. provides four different source models for positions 0, 3, 6, and 12 mm from the tip wall to account for these dwell position‐dependent anisotropy factors. The vendor provided the anisotropic factors for the studied source and applicator combinations. Our secondary objective is to quantify the anisotropy factors by measurements and compare them to the vendor‐supplied source models.

### 1.C Dosimetry protocols

Dose to water dosimetry protocols for brachytherapy have historically used the AAPM's Task Group 43; Dosimetry of Interstitial Brachytherapy Sources.^10^ Modifications were implemented in Task Group 43u; Update of AAPM Task Group No. 43: A revised AAPM protocol for brachytherapy dose calculations.^11^ A modified version of TG‐43 has been implemented to formalize the dosimetry parameters for the Xoft Inc. x‐ray source.^6^, ^12^ Rivard et al. recommended eq. (1) for calculating the 2D dose rate distribution for a Xoft Inc. source assuming point source geometry where **S**_**K**_ is the air‐kerma strength of the source, **Λ** is the dose rate constant in water, **g**_**p**_(**r**) is the radial dose function, and **F**(**r**, **θ**) is the 2D anisotropy function.^6^

(1) $$\dot{D}\left(r,\theta \right)=S_{K}\cdot \Lambda \cdot {\left(\frac{r_{o}}{r}\right)}^{2}\cdot g_{p}\left(r\right)\cdot F\left(r,\theta \right).$$

In our paper, we quantize the polar anisotropy factor for different source positions using radiochromic film and compare them to their respective vendor‐supplied source models. Accurate modeling of the parameters in eq. (1), including the anisotropy factor, enables the clinician to make informed decisions about a treatment plan and potentially increase patient outcomes. To verify the point source model specified by Rivard et al. and identify any manufacturing defects with the tandem and source, we analyzed the azimuthal anisotropy.

### 1.D Energy response of radiochromic film

It is well known that EBT3 radiochromic films present significant energy response dependence in the orthovoltage x‐ray energy range.^13^ Villarreal‐Barajas and Khan has observed a 20% underresponse for 70 kVp beams with respect to Cobalt‐60 clinical beams.^10^ To alleviate this effect and the inherent energy spectrum changes, we irradiated our calibration films at conditions that were similar to the measurements that we were interested in. The third objective of our study is to evaluate the radiochromic film response and accuracy at low kV energy.

## 2 Materials and methods

### 2.A Measurement uncertainty analysis

Uncertainty analysis was completed in accordance with the NIST Technical Note 1297.^14^ Table 1 summarizes the uncertainties associated with the measurements in this paper. Individual uncertainties for each color channel were found to be similar for all parameters, except calibration curve fitting, and the maximum uncertainty is reported representing a “worst‐case scenario”. Uncertainties associated with the calibration curve fitting are reported for two different scenarios in an attempt to minimize uncertainty. The first scenario minimizes the uncertainty by utilizing only the appropriate dose range in the calibration curve for each color channel; red channel for 0–6 Gy and the green channel for 6–35 Gy. The second scenario is calculating the uncertainty for each color channel for all dose ranges in the calibration curve. Film orientation is not used for calculating total uncertainty due to all films being scanned in the same orientation. Taking the quadrature sum of all uncertainties is used for calculating the total uncertainty.

**Table 1:** Dose uncertainties

Parameter	Uncertainty (%)
Film scanner	
Scan time	0.2
Reproducibility of response	0.2
Homogeneity	0.4
Positioning	0.1
Film	
Film reproducibility of response	0.3
Film homogeneity	0.2
Film uniformity	0.4
Film calibration curve fitting	3.8–7.8
Film positioning	5.6
Film orientation	7.5
Source	
Output	2.0
Energy spectrum	3.8
Total uncertainty^a^	8.1–10.5

a Film orientation not used for calculation.

### 2.B Radiochromic film

The radiochromic film used in this study was Gafchromic EBT3 (Gafchromic, International Specialty Products, Wayne, NJ) lot# 12291502. Some solid phantoms give nonnegligible dose differences compared to water,^15^ so all measurements were conducted in water to reduce the uncertainty of using water equivalent phantoms. Aldelaijan et al. studied the impact of water immersion for radiochromic film and the resulting change in optical density, and reported that the maximum anticipated dose error for a 2 × 2 in Ref. 2 film immersed for 0.5 h and scanned 24 h postimmersion was 0.6 cGy.^16^ All films analyzed will be immersed in water for the minimum amount of time possible and for no longer than 0.5 h. According to Marroquin et al., Gafchromic film is more sensitive in the dose range of 0–6 Gy when scanning with the red channel, whereas from 6 to 35 Gy, the response is more sensitive scanning with the green channel.^17^ For doses greater than 35 Gy, the sensitivity in the response of the film is maximized if the film is scanned with the blue channel. Therefore in this study, we verified that the dose range suggested by Marroquin et al. was valid and present the errors associated with analyzing with an incorrect color channel.

### 2.C Scanning protocol

An Epson Perfection v750 desktop flatbed scanner (US Epson, Long Beach, CA) and supplied software, Epson Scan, was used for all film scanning. A film scanning protocol was implemented to reduce the uncertainty of the scanning system. Five empty scans were conducted prior to any measurement scans to warm up the scanning light and detectors. The scanner surface and all films were cleaned prior to scanning with a lint free rag and alcohol for consistency. The films were handled exclusively with latex gloves, and care was taken to avoid any warping of film. All films were scanned in the central region of the scanner, away from the calibration area of the scanner. Typical natural curvature of film at scanning can give rise to a maximum height of 1 to 2 mm above scan plane and may introduce dose errors of 1% to 4%.^18^ To alleviate this error, a specialized film holder was constructed to make the films flush with the scanner plane. All films were scanned in the landscape orientation and transmission mode at 150 dpi resolution, 48 bit RGB (16 bits per color channel), no color corrections, and saved in tiff format. To test the performance of the scanner and the degradation of image quality due to scanner run‐time, an un‐irradiated piece of film was scanned 20 consecutive times and analyzed. To test the integrity of the scanner in transmission mode an un‐irradiated piece of film was scanned and pixel values were analyzed in portrait and landscape mode. Before the films were to be irradiated, they were prepared by cutting a large piece of film (10 × 8 in) into (5 × 4 in) pieces. A special tool was used to cut a hole in the center of the film for the azimuthal anisotropy measurements. Due to de‐lamination of film layers, which can lead to erroneous results, the first four millimeters from any cut and film border was not used for analysis. The films were then individually scanned using the aforementioned scanner parameters for background readings. After irradiation, the films were cleaned and placed in a dark room for a minimum of 12 h to allow for stabilization.

### 2.D Film analysis

After scanning the films, the tiff files were imported into a free image processing and analysis software, ImageJ (http://https://imagej.nih.gov/ij/), and pixel values were inverted. A 3 × 3 average pixel smoothing filter was applied to all background readings. A background pixel value was obtained in the area of interest using the mean pixel value of a 5 mm square located in the area of interest. This was repeated for all three color channels. For irradiated films, a 3 × 3 average pixel smoothing filter was applied and background pixel values were subtracted from irradiated pixel values. A color channel‐dependent pixel value calibration curve was then applied to the respective color channel to be analyzed.

### 2.E Calibration curve

**Figure 2:**
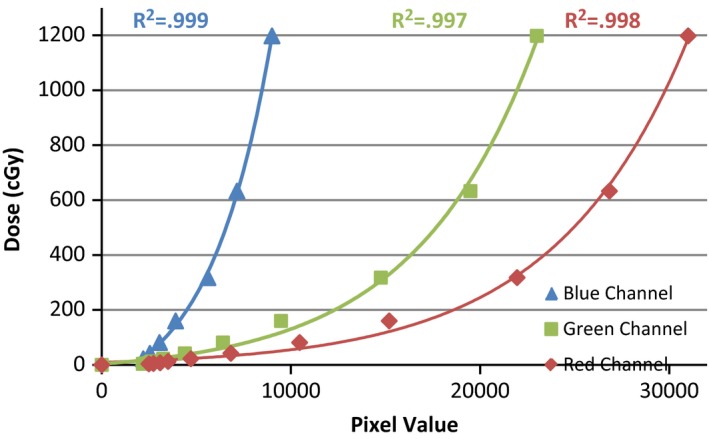
Calibration curve for the three color channels relating pixel value to dose to water (cGy).

**Figure 3:**
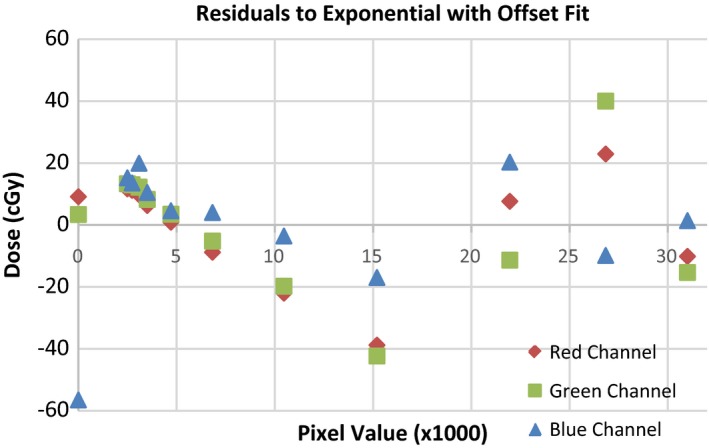
Residuals to exponential with offset fit.

Calibration films were irradiated in water, orthogonal to the long axis of the tandem at a set distance of 1.3 cm from the tip of the tandem. Films were initially setup abutting the tandem, and mechanically moved the specified distance using an IBA Blue Helix scanning tank (IBA Dosimetry, Schwarzenbruck, Germany) with 0.1 mm accuracy. Calibration films were irradiated under these conditions due to significant energy response of radiochromic film in the orthovoltage energy range as reported by Villarreal‐Barajas and Khan.^9^ Calibration films were irradiated at varying beam‐on times, correlating to 3.7 cGy up to 1198.1 cGy. The films were then scanned using the aforementioned protocol. The central area of irradiated films was determined by measuring profiles in the x and y direction and locating the maximum pixel value in the respective profile. A 2 mm diameter circle was centered at the intersection of the profiles and the mean pixel value in the circle was extracted from the software for all three color channels. A calibration curve was then plotted correlating pixel value to dose, with an added point to signify zero dose. A curve fitting program built into ImageJ was used to determine a curve of best fit (Fig. 2). Several different curves were analyzed and the best fit was found using an exponential with offset curve. The residuals to the exponential with offset calibration curve are displayed in Fig. 3. Corresponding *R* ^2^ values are displayed in Fig. 2 for their respective color channels.

### 2.F Source and dose verification

**Figure 4:**
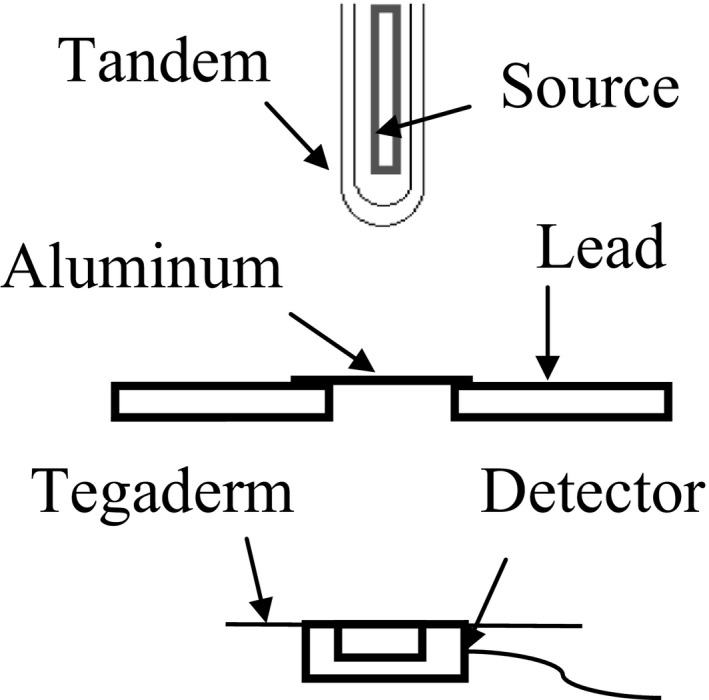
HVL setup.

Dose values that were used for the calibration curves were measured in water with a PTW 34013 parallel plate ionization chamber (PTW, Freiburg, Germany) that was waterproofed using Tegaderm, (3M Health Care, St Paul, MN) and PTW Unidos Webline electrometer. The ionization chamber was calibrated absorbed dose to water from an ADCL laboratory, with correction factors provided to account for different beam qualities. The correction factors varied by as much as 3.8% with a 3.3% uncertainty for the beam qualities of interest. Fulkerson et al. described in detail about the shortcomings of the chamber model that we used for dose verification, noting that her research applies to air‐kerma calibration factors and a TG‐61 approach to determining skin dose.^19^ To accurately model the beam in air and measure the attenuation of the Tegaderm, we used a modified TG‐61 HVL method to measure the HVL for the bare source and the source in tandem.^20^ Altering the energy spectrum with aluminum provided us the ability to verify the attenuation of Tegaderm at different energy spectrums. The ionization chamber was positioned 30 cm away from the source, with a lead aperture placed midway between the source and the detector to collimate the beam (Fig. 4). Aluminum was placed midway between the source and detector for determination of HVL. The chamber was then irradiated with and without Tegaderm; for the bare source and the source placed in the tandem. HVL was determined for both these scenarios. Attenuation of Tegaderm was measured for both scenarios; with and without aluminum.

Dose verification for the calibration curve was measured in water with the PTW 34013 ionization chamber at the same distance and orientation as the film. The chamber was positioned using the same technique as previously described for the calibration films and irradiated for varying times. Dose readings were corrected for temperature and pressure and compared to the expected values from the treatment planning system, taking into account the 2 s ramp‐up time of the electronic brachytherapy source as recommended by the manufacturer.

### 2.G Polar anisotropy

2D Polar anisotropy values assuming a point source were determined using eq. (2), where $\dot{D}\left(r,\theta \right)$ is the dose rate as a function of radius and angle, and $\dot{D}\left(r,\theta_{0}\right)$ is the dose rate at 1 cm on the transverse plane.

(2) $$F\left(r,\theta \right)=\frac{\dot{D}\left(r,\theta \right)}{\dot{D}\left(r,\theta_{0}\right)}$$

**Figure 5:**
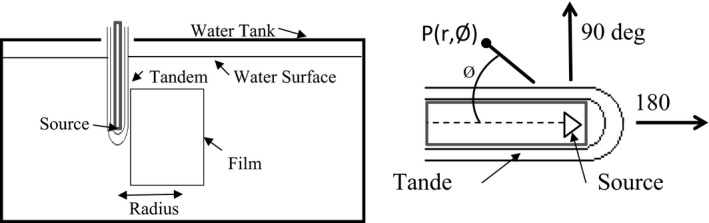
Experimental setup and coordinate systems for polar anisotropy measurements

To determine the polar anisotropy values with radiochromic film, we irradiated two sets of film to different dose levels. The high‐dose set was to receive 1150 cGy and the low‐dose set was to receive 290 cGy, both at 1 cm from the long axis of tandem. The film was placed parallel and abutting the tandem in a specialized film jig. The film was marked, and this mark was aligned to a set position on the tandem. Using the mark on the film allowed us to accurately correlate the spatial coordinates of the source when analyzing the films. The angles were measured from the proximal end of source (Fig. 5). Polar anisotropy values were then compared to vendor‐supplied values.

### 2.H Azimuthal anisotropy

**Figure 6:**
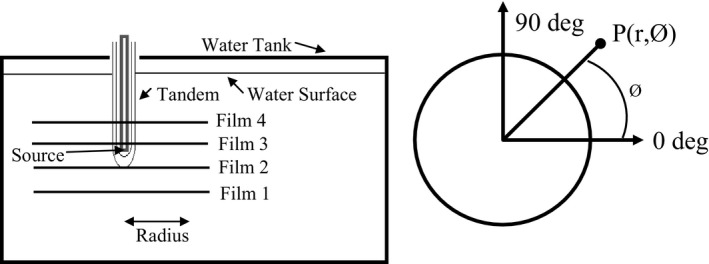
Experimental setup and coordinate systems for azimuthal anisotropy measurements.

For azimuthal anisotropy measurements, a specialized film holder was constructed which allowed us to use a four film array, with the film plane orthogonal to the long axis of the tandem (Fig. 6). Each film was separated by 1 cm. The film holder was lowered into the water tank and the tandem was inserted through the holes that were previously cut into the center of the films. The central point of the film was determined and dose was calculated at varying radii and angles around this central point according to the coordinate system as illustrated in Fig. 6. Delivered dose at 1 cm to film 3 was approximately 500 cGy and the red channel was used for analysis. Azimuthal anisotropy was calculated by first averaging the dose at a specified radius over all angles, and normalizing each individual angle to the average. Expected azimuthal anisotropy value is one.

## 3 RESULTS

### 3.A Film scanner

**Figure 7:**
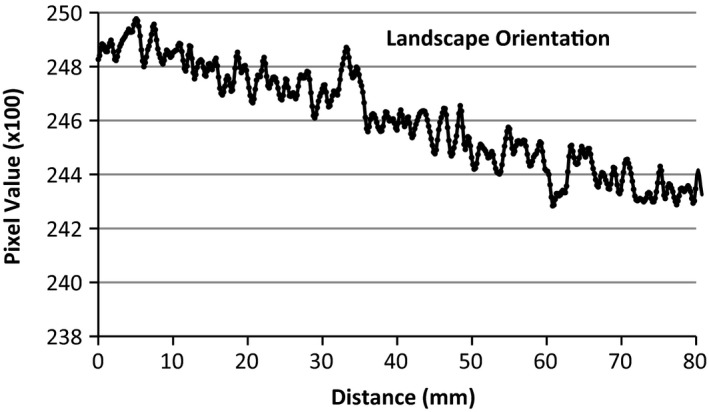
Pixel values in landscape orientation.

**Figure 8:**
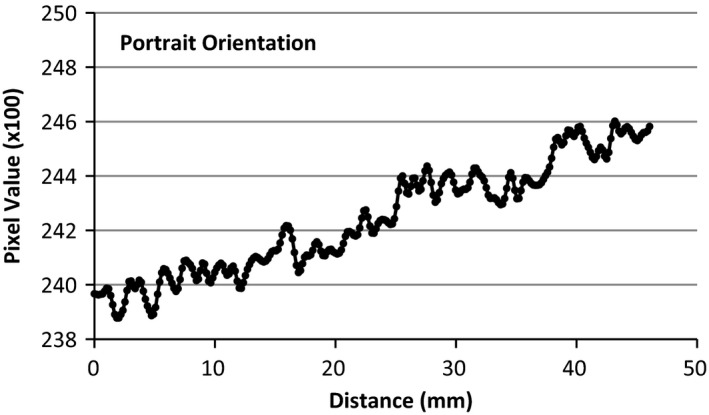
Pixel values in portrait orientation.

The discrepancy from the first film scan to the last film scan was found to be <1%, confirming that there was no degradation in image quality due to scanner run‐time. Figure 7 displays the pixel value as a function of distance across the landscape orientation, with a relative standard deviation of pixel values to be 0.77%. Figure 8 shows the corresponding portrait orientation and the relative standard deviation is 0.83%. On both figures, the origin is defined by the central area of the scanner. These results are within tolerances specified by the film manufacturer and previous published work.^18^

### 3.B Source and dose verification

The average HVL for the bare source was 0.49 mmAl, which agrees well with the measurements by Liu et al.; who measured the HVL at 0.5 ± 0.2 mmAl, using a similar, modified TG‐61 setup.^8^ Table 2 shows that the attenuation by Tegaderm are all less than 0.4% for the four different methods accounting for changing energy spectrum.

**Table 2:** Tegaderm attenuation

	Bare source	Bare source HVL	Source in tandem	Source in tandem HVL
Percent attenuation of tegaderm	0.38%	0.31%	0.29%	0.18%

Measured dose at the specified distance agreed with the treatment planning system expected dose to within 2%. Dose measurements were calculated using the TW‐30 (0.43 mmAl HVL) beam quality calibration factor provided by the calibration laboratory. Determining the exact beam quality at our depth is outside the scope of this research. Adjusting the calibration factor to either the TW‐50 (1.10 mmAl HVL) or TW‐15 (0.1 mmAl HVL) varies the absolute dose output by a maximum of 2%.

### 3.C Polar anisotropy

**Figure 9:**
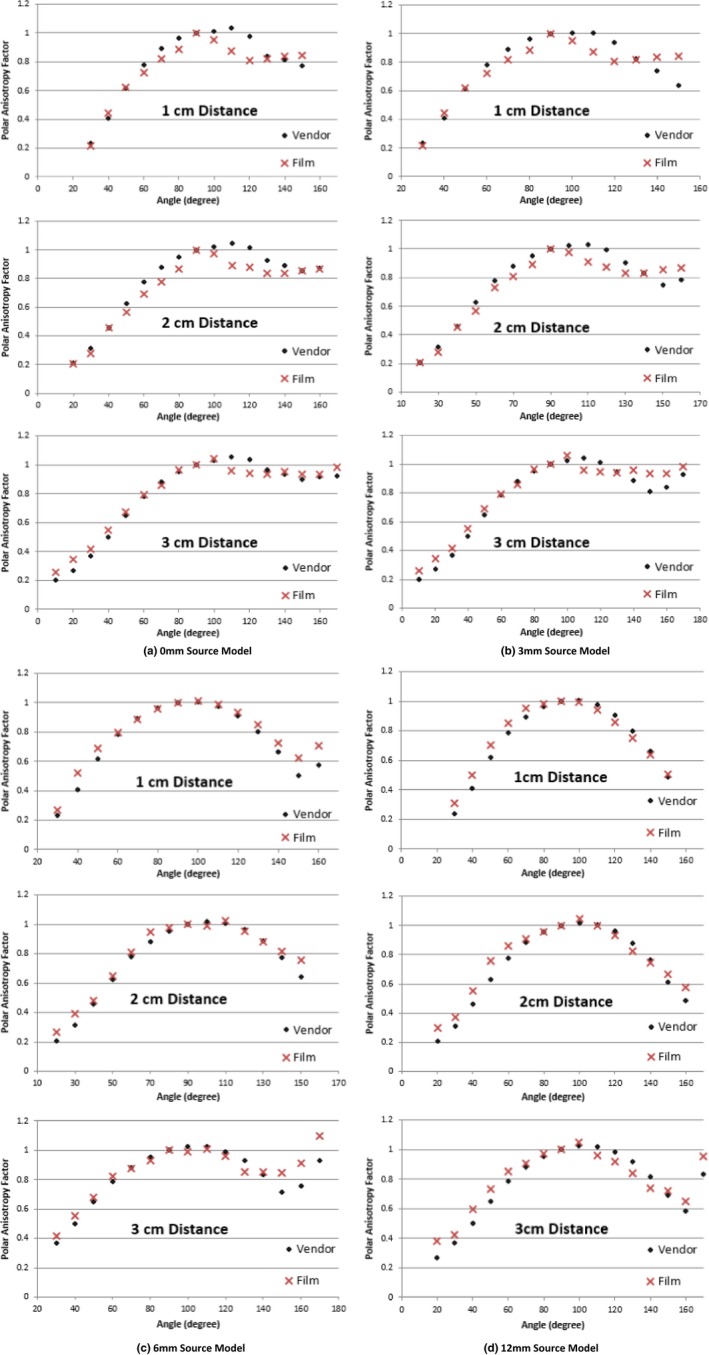
Comparison of polar anisotropy factor between film measurements and vendor‐supplied source model at three distances, using low‐dose film sets, analyzed by the red channel for the (a) 0 mm source model, (b) 3 mm source model, (c) 6 mm source model, (d) 12 mm source model.

As shown in Fig. 9, the measured polar anisotropy functions are compared to the vendor‐supplied models at three different distances using the low‐dose film set analyzed with the red channel for all four source models. The measured anisotropy factors showed relatively good agreements with vendor‐supplied models. Discrepancies were found where the photons were traversing the most oblique and thick part of the tandem. We found with this low‐dose method that the tandem was attenuating the photons more than expected for all source models from 100° to 130° and the majority of distances (Fig. 9). This angle correlates to where the obliquity is the greatest in the tandem. Discrepancies were also found for all source models and distances from 130° to 160°, where the dome tip was the thickest.

**Figure 10:**
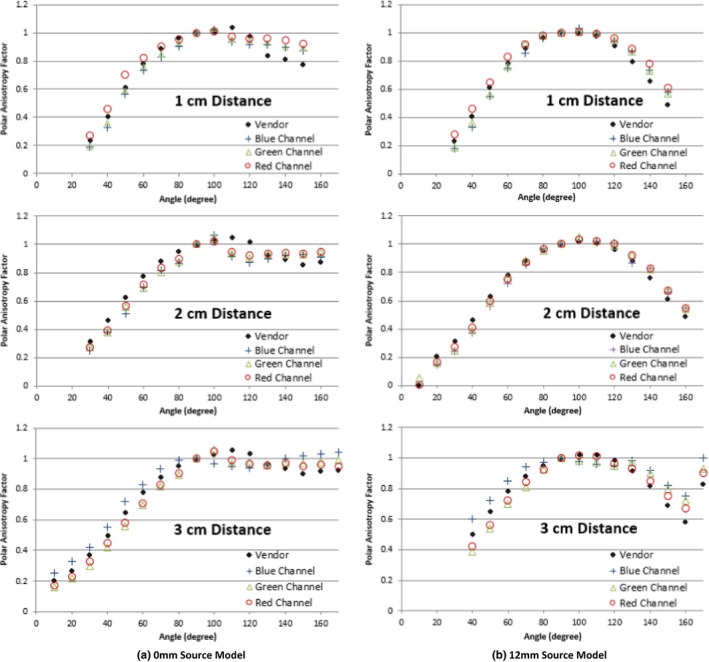
Comparison of polar anisotropy factor between film measurements and vendor‐supplied source model at three distances, using high‐dose film sets, analyzed using all three color channels for the (a) 0 mm source model and (b) 12 mm source model.

Figure 10 shows similar comparisons for the 0 and 12 mm source models except using the high‐dose film set and analyzed with all three channels. Expected dose values at 1, 2, and 3 cm are 1150, 256, and 99.0 cGy, respectively. Following the protocol for analyzing the film with the most sensitive color channel; the 1 cm distance should be analyzed with the green channel and the 2 and 3 cm distance should be analyzed with the red channel. As discussed previously in Methods and Materials, our findings agree with Marroquin et al. for selecting the most sensitive color channel for respective dose range. The measured anisotropy functions showed the same pattern as the low‐dose films for all three color channels for the 0 mm source model [Fig. 10(a)]. At the most oblique angles, 100°–130°, the source model was under compensating for attenuation and the vendor‐supplied polar anisotropy factor was greater than measured. Where the photons were having to traverse the thick part of the dome tip, the source model was over compensating for attenuation and the vendor‐supplied polar anisotropy factors were less than measured. The measured 12 mm source model showed good agreement at all distances for their respective color channel, but deviated at the most oblique angles, due to the source model either over or undercompensating. A summary of the average and maximum percent error for each parameter is displayed in Table 3. When using the appropriate color channel for analysis, the maximum percent error for all sources and distances falls outside of our measurement uncertainty, while the mean percent error is within our margin of uncertainty.

**Table 3:** Polar anisotropy percent error for high‐dose film set (%)

	0 mm source model						12 mm source model					
	Red channel		Green channel		Blue channel		Red channel		Green channel		Blue channel	
	Mean	Max	Mean	Max	Mean	Max	Mean	Max	Mean	Max	Mean	Max
1 cm distance	8.3	19.4	7.3	18.8	8.9	19.1	8.6	24.7	7.1	20.9	8.4	23.1
2 cm distance	7.3	15.2	8.1	17.4	9.4	23.9	6.6	20.6	7.3	23.1	8.9	27.9
3 cm distance	6.6	15.4	9.1	20.4	9.9	24.4	6.1	16.2	10.0	23.3	10.6	28.4

### 3.D Azimuthal anisotropy

**Figure 11:**
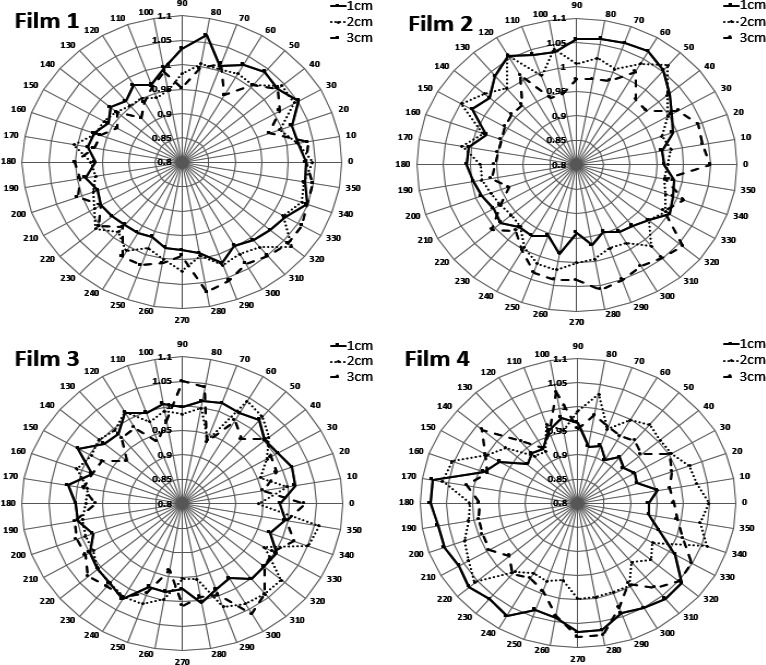
Azimuthal anisotropy factors along the tandem at three distances from the tandem for four film positions. Please refer to Fig. 6 for the experiment setup.

Figure 11 shows the azimuthal anisotropy factors for four positions, as illustrated in Fig. 6, along the tandem at three distances from the tandem with the source at the most extended distance. The average relative standard deviation over all angles and distances for all four films was 3.65% and the maximum relative standard deviation for individual angles averaged over four films was 5.07%. The average relative standard deviation at 1, 2, and 3 cm was 3.87%, 3.12%, and 4.04%, respectively. The maximum deviation was found to be less than our measurement uncertainty.

## 4 DISCUSSION

A quality assurance protocol should be conducted for all new brachytherapy applicators to be implemented for clinical use to identify any manufacturing defects within the construction of the device. Due to deterioration of electronic brachytherapy sources, all sources should be verified upon receipt and at regular intervals over the life of the source.

### 4.A Scanner

The performance of our scanning system was first extensively studied prior to all the measurements and the main tests were reported in the result section. Several other tests were investigated such as total scanner on‐time, resolution, transmission vs reflective, lateral response, film orientation, and film positioning. They were compared to the literature and showed that the scanning system performed as expected (data are not shown due to paper length limit).

### 4.B Radiochromic film

Brown et al. found an underresponse of relative sensitivity (defined as netOD_xxkV_/netOD_4MV_) ranging from 0.97 to 0.99 for monochromatic x rays of 25, 30, and 35 kV at varying dose levels (50–200 cGy) in a PMMA phantom.^21^ Masillon et al. found an underresponse of relative sensitivity (defined as netOD_50kV_/netOD_6MV_) of 0.90, 0.89, and 0.93 for the red, green, and blue channels, respectively, at 100 cGy for a 50 kVp beam (HVL = 0.77 mmAl) using an in‐air setup.^22^ Villarreal‐Barajas and Khan reported underresponse of relative sensitivity (defined as netOD_xxkV_/netOD_CO‐60_) of approximately 20% for beams with effective energies of ~32 and ~38 kV (70 kVp and 100 kVp), contradicting Brown et al. and Masillon et al.^13^ Villarreal‐Barajas and Khan also concluded an underresponse of relative sensitivity for the 100 kVp beam of 0.83, 0.83, and 0.76 for the red, green, and blue channel, respectively, and an underresponse of relative sensitivity for the 70 kVp beam of 0.79, 0.8, and 0.74 for the red, green, and blue channel, respectively. This indicates that there is an underresponse between 70 and 100 kVp beams.

The change in energy spectrum with depth and interfaces has the potential to introduce errors into any calibration curve. Due to the conflicting literature and changing energy spectrum with depth, it is recommended to calibrate the films at the desired dose range and energy spectrum, as performed in this study. Readers are referred to the section “Film Analysis” for the details on converting pixel value into dose for this study.

### 4.C Polar anisotropy

We found a deviation from the measured and vendor‐supplied polar anisotropy factor at the oblique angles where the photons traverse the maximum distance through the titanium. These discrepancies were verified using different calibration films, different dose ranges, and altering the position of the point source. The source of these discrepancies could be due to manufacturing defects within the source and/or tandem. Please note that it is beyond the scope of this paper to identify the cause of this discrepancy.

### 4.D Azimuthal anisotropy

The tandem was noticed to be attenuating the source preferentially from 90° to 180° for the majority of films and distances, but within our margin of uncertainty. A positioning error of 1 mm could introduce up to a 10% error in dose, and standard deviation values reported are within values reported by Rivard et al.^6^ Another source of uncertainty in the azimuthal anisotropy measurements could be the asymmetric photon production within the source.^6^

## 5 CONCLUSION

We systematically investigated polar and azimuthal anisotropy factors for a new electronic brachytherapy source and tandem by film measurements and compared to the vendor‐supplied source models. Radiochromic film response and accuracy was also evaluated. We concluded that the vendor‐supplied anisotropy factor was adequate for clinical use at majority of the angles. A rigorous quality assurance method for new electronic brachytherapy sources and applicators, along with complete knowledge of all dosimetric measuring tools, should be implemented for all parts of the verification and commissioning process.

## CONFLICTS OF INTEREST

The authors have no relevant conflicts of interest to disclose.
